# P21 activated kinase‐1 (PAK1) in macrophages is required for promotion of Th17 cell response during helminth infection

**DOI:** 10.1111/jcmm.16050

**Published:** 2020-10-30

**Authors:** Hao Chang, Kai‐Yue He, Chen Li, Yang‐Yue Ni, Mai‐Ning Li, Lin Chen, Min Hou, Zikai Zhou, Zhi‐Peng Xu, Min‐Jun Ji

**Affiliations:** ^1^ Center for Global Health Nanjing Medical University Nanjing China; ^2^ Department of Pathogen Biology Jiangsu Province Key Laboratory of Modern Pathogen Biology Nanjing Medical University Nanjing China; ^3^ Shanghai Key Laboratory of Psychotic Disorders Shanghai Mental Health Center Shanghai Jiao Tong University School of Medicine Shanghai China

**Keywords:** IL‐6, macrophage, P21 Activated Kinase‐1, Th17 cell, Treg cell

## Abstract

CD4^+^T cells differentiate into distinct functional effector and inhibitory subsets are facilitated by distinct cytokine cues present at the time of antigen recognition. Maintaining a balance between T helper 17 (Th17) and regulatory T (Treg) cells are critical for the control of the immunopathogenesis of liver diseases. Here, by using the mouse model of helminth *Schistosoma japonicum* (*S* *japonicum*) infection, we show that the hepatic mRNA levels of P21‐activated kinase 1 (PAK1), a key regulator of the actin cytoskeleton, adhesion and cell motility, are significantly increased and associated with the development of liver pathology during *S* *japonicum* infection. In addition, PAK1‐deficient mice are prone to suppression of Th17 cell responses but increased Treg cells. Furthermore, PAK1 enhances macrophage activation through promoting IRF1 nuclear translocation in an NF‐κB‐dependent pathway, resulting in promoting Th17 cell differentiation through inducing IL‐6 production. These findings highlight the importance of PAK1 in macrophages fate determination and suggest that PAK1/IRF1 axis‐dependent immunomodulation can ameliorate certain T cell–based immune pathologies.

## 1 INTRODUCTION

The immune systems, which mainly consist of T cells, B cells and NK cells, are responsible for the maintaining of host homeostasis, adaptive immune responses providing protection against disease and specially infectious disease.^1^ In particular, CD4^+^ T cells different subsets were essential for immune responses during host defence against pathogen infection and inflammatory diseases,^2^, ^3^, ^4^, ^5^, ^6^, ^7^ mainly including Th1, Th2, Th17 and Treg cells. The signals promoting differentiation of naive CD4^+^ T cells into a particular T helper cell subset are provided by distinct cytokines through activating antigen‐presenting cells (APCs).

P21‐activated kinases 1 (PAK1), which was highly expressed in antigen‐presenting cells (APCs),^8^, ^9^ contained conserved six serine‐threonine kinases and involved in cellular apoptosis, proliferation and migration.^10^, ^11^, ^12^ PAK1 has been reported to play a vital role in variety of disorders such as cancer, Alzheimer's disease, diabetes, and neurofibromatosis.^13^ Recent studies have implicated that PAK1 was overexpressed in the inflammation‐related disease and promoted cell activation pathways expression involved in NF‐κB signalling.^12^, ^14^, ^15^ However, the way in which PAK1 mediates its adaptive cellular effects during pathogen infections remains unclear.

Schistosomiasis (bilharzia) was an important parasitic disease that affects more than 250 million people worldwide and can induce a complex immune response.^16^ Both macrophages and CD4^+^ T cells were critical for the regulation of the hepatic granulomatous and fibrosis response.^17^ During the acute stage of *Schistosoma japonicum* (*S japonicum*) infection, the classical activation of macrophage and Th1 response are induced by the worm antigen stimulation.^18^ After the egg production, the immune responses manifest a striking shifted from classical activated macrophages to robust alternative activation of macrophage and three main effector CD4^+^ T‐cell types (Th1, Th2 and Th17 cells),^17^, ^19^ which contributed to the development and regulation of granulomatous inflammation and fibrosis response.^20^, ^21^ Therefore, schistosome infection is an excellent model for studying the interactions between innate and adaptive lymphocytes that characterize to figure out the mechanism of CD4^+^ T cells immune response.^17^, ^22^

In this study, we explored the role of a key regulator in CD4^+^ T cells fate determination using the murine *S japonicum* infection model. For the first time, we found that macrophage PAK1 can induce CD4^+^ T cells to differentiate into Th17 cells through IRF1/IL‐6 axis. Our findings provide new insights into controlling the T cell–based immune pathologies and suggest new strategies to manipulate these cell lineage decisions in order to treat diseases associated with Th17/Treg imbalance.

## 2 MATERIALS AND METHODS

### 2.1 Ethics statement

The execution of animal experiments was strictly observed with the Regulation for the Administration of Affairs Concerning Experimental Animals (1998.11.1). The ethics (IACUC‐1905065) were approved by the Institutional Animal Care and Use Committee (IACUC) of Nanjing Medical University.

### 2.2 Experimental mice and parasites

Six‐week‐old C57BL/6 female mice were purchased from Animal Core Facility of Nanjing Medical University. PAK1‐deficient mice were kind gifts from Dr Zhengping Jia (The Hospital for Sick Children, Canada).^23^, ^24^, ^25^ The sex‐ and age‐matched mice were bred in specific pathogen‐free conditions. *Oncomelania hupensis* that possessed *S* *japonicum* cercariae were purchased from the Parasitic Disease Prevention and Research Institute of Jiangsu Province. Six weeks old C57BL/6 female mice were infected with 12 *S japonicum* cercariae via shaved abdomen. The mice were killed at 3, 7 and 11 weeks for detecting the dynamic mRNA levels of PAK1, while the time point of 8 weeks post‐infection was chosen for detecting the hepatic pathology and the immune‐related factors analysis.

### 2.3 Preparation of soluble egg antigen

Schistosome soluble egg antigen (SEA) was prepared based on methods previously described with some modifications.^26^ Briefly, New Zealand rabbits were infected with about 1500 *S japonicum* cercariae by the abdomen. The rabbits were killed 6 weeks after infection. Parasitic eggs were isolated from the liver by enzymatic digestion using 0.05% collagenase B (Sigma‐Aldrich, St. Louis, USA), the eggs were suspended in PBS and freeze‐thawed several times and were centrifuged at 4°C and 15 000 × g for 30 min, the suspensions were collected and sterilized with a 0.22 μm filter. The endotoxin in suspensions was removed by ToxOut™ Rapid Endotoxin Removal Kit (Biovision, Milpitas, USA). Then, the residual endotoxin in the SEA solution was detected by using the Pierce LAL Chromogenic Endotoxin Quantitation Kit (Thermo Scientific, Rockford, USA) according to manufacturer's recommendations. Finally, the concentration of SEA was tested with a bicinchoninic acid (BCA) Protein Assay Kit (Sigma‐Aldrich).

### 2.4 Quantitative real‐time PCR

For real‐time quantitative PCR analysis, the total RNA was isolated by TRIzol (Invitrogen, Carlsbad, CA). RNA was reversed into cDNA using HiScript II Q Select RT SuperMix for qPCR (Vazyme, Nanjing, China). Amplification was analysed by AceQ qPCR SYBR Green Master Mix (Vazyme). Relative expression was calculated using the 2^−ΔΔct^ method and normalized to GAPDH. Gene expression was quantified using the LightCycler® 96 System (Roche Life Science, Basel, Switzerland). The primers are shown in the Table S1.

### 2.5 Lymphocytes preparation

Spleen lymphocytes were prepared as the previous study reported.^27^ Briefly, the spleen was grinded in the complete RPMI 1 × 1640 medium (Gibco, Palo Alto, USA) with 10% FBS and 1% PS, the red blood cell (RBC) was lysis by erythrocyte lysis buffer (0.15 M NH_4_Cl, 10 mM KHCO_3_, 0.1 mM Na_2_EDTA, pH7.2), then the suspensions filtered through a 70 μm nylon mesh, and washed by PBS with 1% FBS and counted through a microscope.

The method of isolated hepatic lymphocytes as previously described with some modifications.^27^, ^28^ Briefly, after anesthetization, the liver was perfused with 10 mL of sterile PBS through the portal vein, and subsequently excised and cut into small pieces, digested in the buffer contained with collagenase IV 5% (Sigma‐Aldrich) and 100 μg/mL DNase I (Roche Life Science) at 37°C for 30 minutes. Then, the liver suspensions were passed through 70 μm nylon mesh and centrifuged at low speed (60 × g, 2 minutes) to remove hepatocytes. The hepatic lymphocytes were separated using 35% Percoll (Sigma‐ Aldrich) by centrifuging at 600 × g for 20 minutes. Lymphocytes were collected and lysis in erythrocyte lysis buffer to remove RBC.

### 2.6 Flow cytometry

The purified spleen and liver cells were counted and their viability assessed using the trypan blue exclusion method.^29^ Then, the absolute cell counts (2 × 10^6^) and suspension were in the complete RPMI 1640 medium. All cells were detected by BD FACSVerse and analysed by FlowJo software (Treestar, Inc, San Carlos, USA).

For Th1/Th2/Th17 analysis, the cells were stimulated for 4 h with 25 ng/mL PMA (Sigma‐Aldrich), 1 mg/mL Ionomycin (Sigma‐Aldrich) and 0.66 μL/mL Golgistop (Sigma‐Aldrich) and cultured at 37°C in 5% CO_2_. The cells were harvested and stained with rat anti‐mouse CD3‐PerCP‐Cy™5.5 (BD Pharmingen, San Diego, USA) and rat anti‐mouse CD4‐FITC (eBioscience, San Diego, USA). After being fixed and permeabilized with FIX&PERM Kit (MultiSciences, Hangzhou, China), the cells were intracellularly stained with rat anti‐mouse IFN‐γ‐PE (eBioscience), rat anti‐mouse IL‐4‐PE (BD Pharmingen) and rat anti‐mouse IL‐17A‐PE (BD Pharmingen).

For Tregs analysis, single‐cell suspension was stained with rat anti‐mouse CD4‐FITC (eBioscience) and rat anti‐mouse CD25‐APC (BD Pharmingen). After being washed, fixed and permeabilized with Fixation/Permeabilization Diluent (eBioscience), then the rat anti‐mouse Fc block (BD Pharmingen) was added into cell suspension and incubated for 15 minutes at 4°C. The cells were intracellularly stained with rat anti‐mouse Foxp3‐PE (BD Pharmingen).

### 2.7 Peritoneal macrophage purification

Peritoneal macrophages were prepared as described previously with some modification.^30^ In brief, normal WT or PAK1‐deficient mice were killed and the peritoneal cavity was lavaged by PBS with 1% foetal serum. Peritoneal exudates cells (PEC) were collected and centrifuged at 400 g for 10 min at 4°C, then resuspended in erythrocyte lysis buffer and centrifuged at 400g for 10 min at 4°C. Cells were resuspended and added into 6‐well plate at the density of 5 × 10^5^ each well. After adherence for 3 h, the medium was replaced to remove the non‐adherent cells. Flow cytometry was used to characterize the purification of isolated cells with PE‐antibody against mouse F4/80 (~98%, rat anti‐mouse, eBioscience).

### 2.8 Cytokine measurement

Peritoneal macrophages (5 × 10^5^) were cultured in a 6‐well plate. Cells were treated with soluble egg antigen (SEA) (25 μg/mL) for 24 h, then the culture supernatants were collected. The concentrations of IL‐6 in the supernatants were analysed by Enzyme‐Linked Immunosorbent Assays (ELISA) according to the manufacturer's instructions (MultiSciences).

### 2.9 CD4^+^ T cells separation and in vitro stimulation

CD4^+^ T cells were purified from the spleen of mice by a negative selection EasySepTM Mouse CD4^+^ T cells Isolation Kit (Stem Cell Technologies, Vancouver, Canada). All experiment operations accord with the manufacturer's recommendations. The purified CD4^+^ T cells (1 × 10^6^) were added into a 6‐well plate and stimulated with SEA (25 μg/mL) for 24 h or co‐cultured with SEA‐stimulated macrophages for 24 h.

### 2.10 Transwell assay

The co‐cultivation transwell model was established based on a previously published method with some modifications.^31^ Briefly, CD4^+^ T cells (1 × 10^6^) were seeded on the bottom of a 12‐well plate containing the 1 mL complete DMEM medium. The peritoneal macrophages (5 × 10^5^) were stimulated with SEA (25 μg/mL) for 24 h, and then the SEA‐activated macrophages were added on the surface of 0.4 μm transwell microporous membrane (BD Biosciences), which were placed into the 12‐well plate to incubate for 24 h. After that, the CD4^+^ T cells were collected and flow cytometry was performed to analyse the expression of different subsets of T cells.

### 2.11 Cell transfection

Transfection of the macrophage cell line (RAW264.7) was carried out as previously described with some modifications.^32^ Briefly, 5 × 10^6^ cells were resuspended in opti‐MEM (Reduced Serum Medium, Gibco, Grand Island, USA) and seeded in 6‐well plate, then transfected with si‐IRF1 (5’‐GGACATTGGGATAGGCATA‐3’) and non‐targeting control siRNA (5’‐UUCUCCGAACGUGUCACGU‐dTdT‐3’) or plasmid overexpressing PAK1 and vector by using Lipofectamine 2000 (Invitrogen, Carlsbad, USA), according to the manufacturer's instructions. Gene knockdown or overexpression was evaluated by Western blot. All small interfering RNAs (siRNAs) and plasmids were purchased from RiboBio (Nanjing, China).

### 2.12 Western blot

Cells were washed three times with sterile cold PBS and lysed with RIPA buffer containing phenylmethanesulphonylfluoride (PMSF). Equal amount of proteins was electrophoresed to SDS polyacrylamide gel and transferred to polyvinylidene fluoride (PVDF) membrane (Merck Millipore, Massachusetts, USA), then the membrane contained proteins was blocked with 5% (w/v) skimmed milk for 1 h at room temperature. The proteins were incubated with the primary antibody overnight at 4°C. After washing three times with TBS‐0.05% Tween 20, the blots were incubated with horseradish peroxidase (HRP)‐coupled goat anti‐rabbit IgG H&L secondary antibody for 2 h at room temperature. The blots were developed with an enhanced chemiluminescence (ECL) system (Merck Millipore), and images were photographed using ChemiDocTM Touch Imaging System (Bio‐Rad, Hercules, USA). The primary antibodies used in this study were rabbit anti‐mouse IRF1 (Cell Signaling Technology, Danvers, USA), rabbit anti‐mouse PAK1 (Cell Signaling Technology), rabbit anti‐mouse phospho‐PAK1(Thr423) (Cell Signaling Technology), rabbit anti‐mouse IL‐6 (Cell Signaling Technology), rabbit anti‐mouse β‐actin (Cell Signaling Technology), rabbit anti‐mouse Histone 3 (Abcam, Cambridge, England), rabbit anti‐mouse phospho‐p65 (Ser536, Abcam) and rabbit anti‐mouse total‐p65 (Abcam).

### 2.13 Immunofluorescence assay

Peritoneal macrophages were fixed with 4% paraformaldehyde for 30 minutes, then washed three times with PBS and permeabilized with 0.03% Triton X‐100 (Sigma‐Aldrich) for 30 minutes at room temperature. Unspecific binding was blocked with 5% Bovine Serum Albumin (Sigma‐Aldrich) for 1 h at room temperature. Peritoneal macrophages were incubated with rabbit anti‐mouse IRF1 (Cell Signaling Technology) (1:200) at 4°C overnight. After washing with 1 × PBST, Peritoneal macrophages were incubated with g goat anti‐rabbit IgG H&L (Alexa Fluor® 647, Abcam) (1:200) for 1 h at room temperature, respectively. Cell nucleus was stained with DAPI Fluoromount (Southern Biotech, Birmingham, USA) and observed under a fluorescence microscope (ZEISS, Imager.A2, Germany).

### 2.14 Statistical analysis

All analyses were carried out with SPSS 21.0 software. Data were shown as the mean ± SEM. Statistical comparisons of two groups were determined using Student's t test. Multiple comparisons were performed by one‐way ANOVA, followed by LSD post‐testing for comparisons between two groups. *P* < .05 was considered significant.

## 3 RESULTS

### 3.1 PAK1 promotes hepatic pathology during *S japonicum* infection

**Figure 1:**
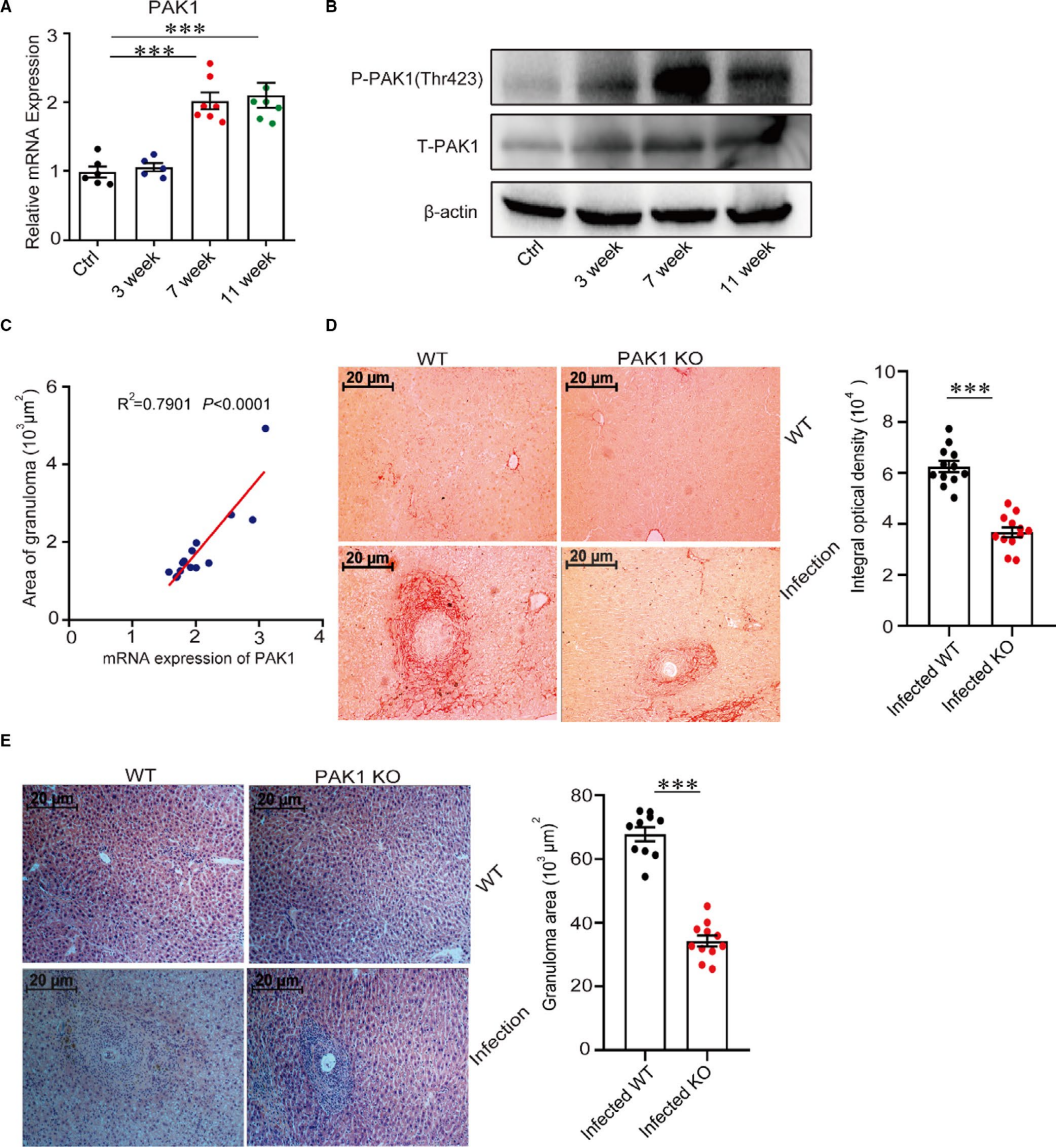
PAK1 promotes hepatic pathology during *S* *japonicum* infection. (A) The PAK1 mRNA levels in the livers of WT and *S japonicum*‐infected mice at indicated time points post‐infection were analysed by real‐time quantitative PCR (n = 7). (B) The dynamic protein expression of phosphorylated PAK1 (Thr423) via Western blot in the livers from *S japonicum*‐infected mice was conducted (n = 5). (C) The correlation was shown between the granuloma size and the expression of PAK1 in the livers from *S* *japonicum*‐infected WT mice. (D, E) Paraffin‐embedded liver sections were stained with H&E or Sirius Red, the original magnification of stained liver sections was 100×, images shown were representative of experiments, scale bar, 20 μm (n = 12). Data were representative of one experiment from two replicate experiments. Data were presented as mean ± SEM. ***P < .001

We firstly investigated the dynamic expression of PAK1, PAK2, PAK3 and PAK4 by real‐time quantitative PCR during *S japonicum* infection. Results showed that there were no significant changes in the hepatic mRNA levels of PAK2 and PAK4 after *S japonicum* infection (Figure S1A). Interesting, the mRNA of PAK1 and protein levels of phosphorylated PAK1 (Thr423) in the liver were significant increased at 7 and 11 weeks’ time point post‐infection by real‐time quantitative PCR and Western blot (Figure 1A, B, Figure S8), suggesting the hepatic PAK1 reaches its peaks in the time period between 7 and 11 weeks. In addition, the hepatic PAK1 had a significant positive correlation with the area of granuloma in the livers from *S* *japonicum*‐infected WT mice (Figure 1C). Although PAK3 was also found to increase in the livers derived from *S japonicum‐*infected mice (Figure S1A), no significant correlation was found with granuloma size (Figure S1B).

To further investigate the roles of PAK1 in the development of liver pathology, PAK1‐deficient mice were used to infect with *S* *japonicum* cercariae. Results showed that the hepatic granuloma size and fibrosis response were largely decreased in PAK1‐deficient mice when compared with WT mice at 8 weeks post‐*S* *japonicum* infection, when the hepatic immunopathological response reaches a peak ^33^ (Figure 1D, E). Altogether, these results demonstrate the highly expressed PAK1 in the liver promotes the hepatic pathology during *S* *japonicum* infection.

### 3.2 PAK1 deficiency promotes regulatory T cells and attenuates inflammation response in vivo

**Figure 2:**
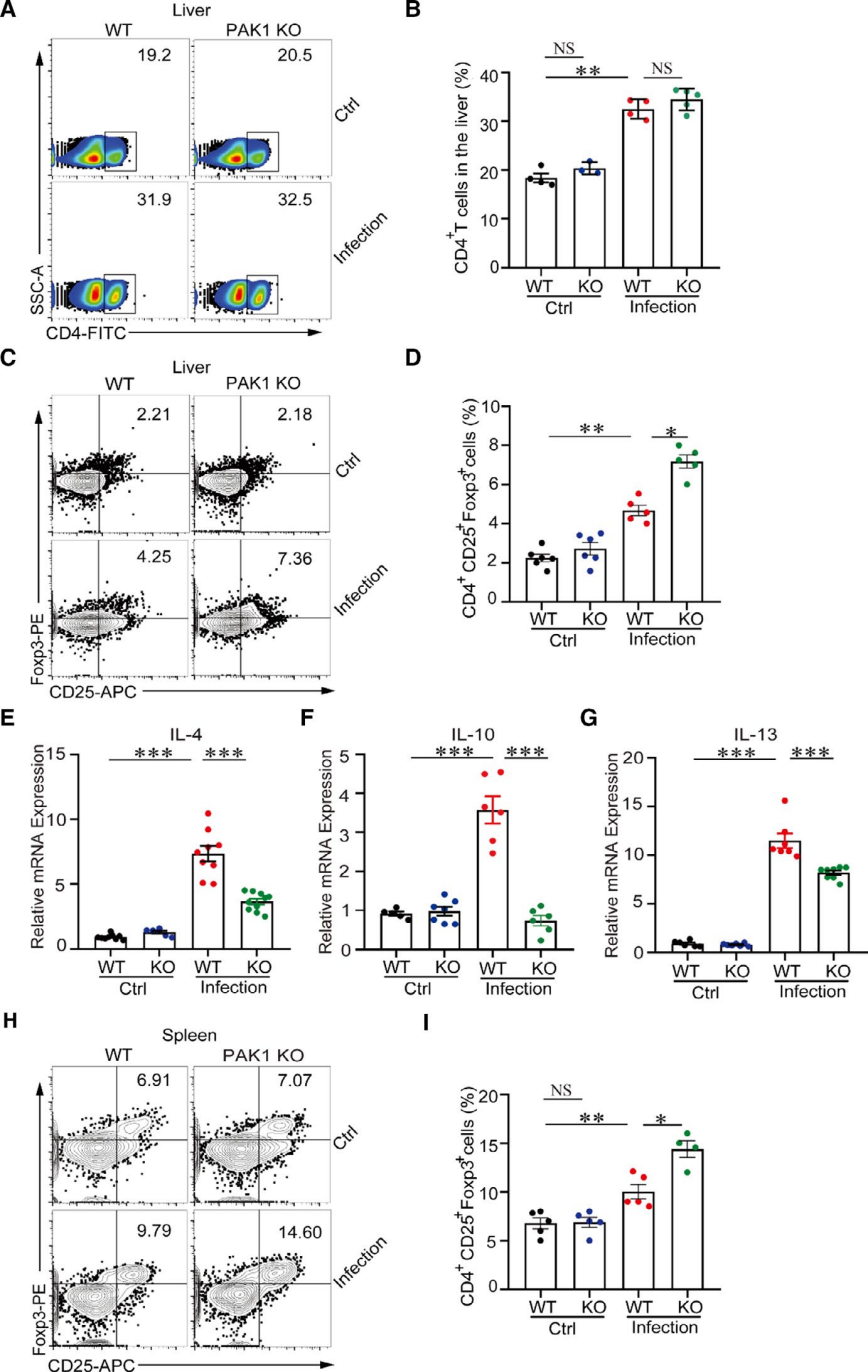
PAK1 deficiency can promote regulatory T cells and attenuate inflammation response in vivo. (A, B) Flow cytometry analysed the expression of CD4^+^ T cells in the livers from WT and PAK1^−/−^ mice with or without *S* *japonicum* infection. (C, D) Flow cytometry analysed the CD4^+^CD25^+^ Foxp3^+^ (Treg) cell population in the hepatic lymphocytes. (E–G) The mRNA levels of IL‐4, IL‐10 and IL‐13 in the livers from WT and *S japonicum*‐infected mice were analysed by real‐time quantitative PCR (n = 10). (H, I) Flow cytometry analysed the proportion of CD4^+^CD25^+^ Foxp3^+^ (Treg) in the spleen. Data were representative one experiment from two replicate experiments each with n = 5 mice per group. Data were presented as mean ± SEM. *P < .05; **P < .005; ***P < .001

As one of the major participants, CD4^+^ T cells played an imperative role in the regulation of immunopathology in schistosomiasis.^17^ Therefore, we investigated whether host CD4^+^ T‐cell subsets were affected by PAK1 deficiency during *S* *japonicum* infection (Figure 2, Figure S2). Although there were no significant differences of CD4^+^ T cells in the livers between WT and PAK1‐deficient mice during *S* *japonicum* infection (Figure 2A‐B), we found that the population of Treg cells, which were needed to control immune responses and to maintain immune homeostasis,^34^ were dramatically increased in the livers from PAK1‐deficient mice when compared with WT mice after *S* *japonicum* infection (Figure 2C‐D). Consistence with this, the hepatic mRNA levels of IL‐4, IL‐10 and IL‐13 were obviously decreased in PAK1‐deficient mice compared with WT mice during *S* *japonicum* infection (Figure 2E‐G).

In addition, the percentages of Treg cells were also found to significantly enhance in the spleen from PAK1‐deficient mice when compared with WT mice under *S* *japonicum* infection (Figure 2H‐I). However, the proportion of Th17 cells (Figures S3A,B) but not Th1 or Th2 cells (Figures S3C,D, E,F) was significantly decreased. Altogether, these results indicate that PAK1 deficiency ameliorates the hepatic inflammation by facilitating the differentiation of Treg cells during *S* *japonicum* infection.

### 3.3 PAK1 deficiency induces an increase in TREG cells but a decrease in Th17 cells in vitro

**Figure 3:**
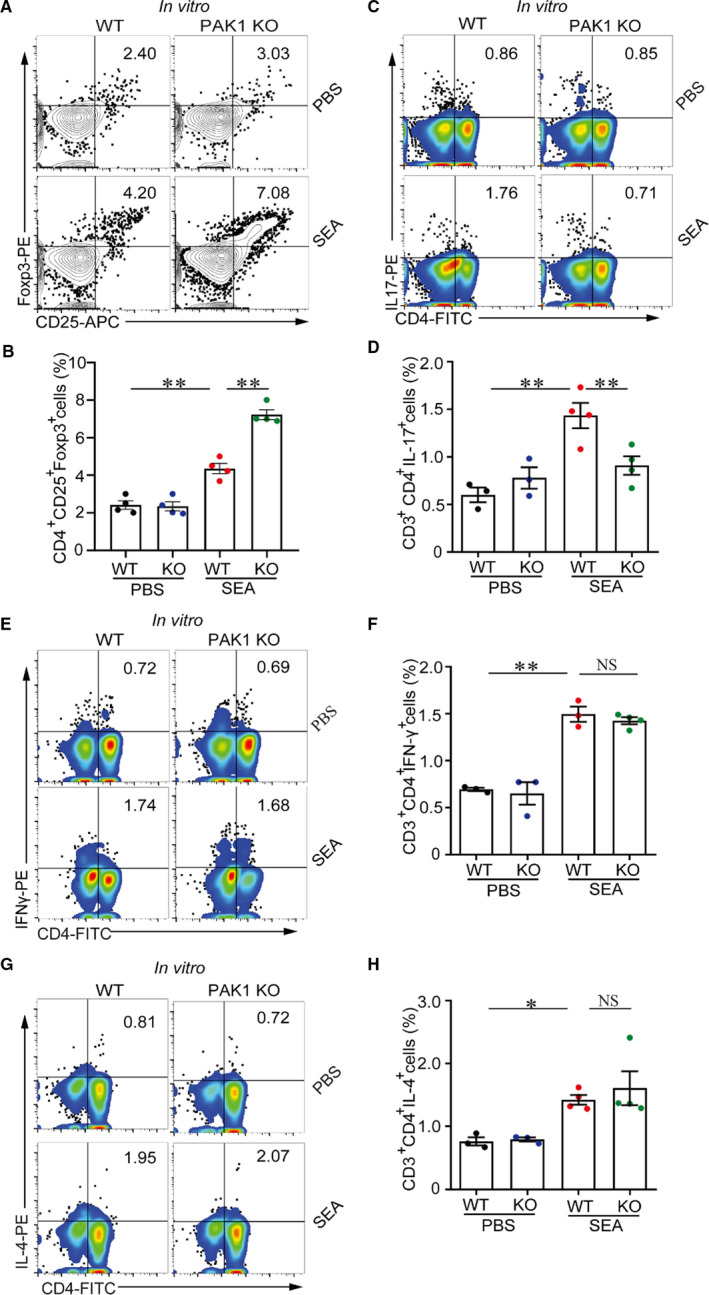
PAK1 deficiency can elevate Treg cells and suppressTh17 cells in vitro. Splenic lymphocytes suspension was isolated from WT mice and PAK1‐deficient mice and analysed the cell phenotype after SEA (25 μg/mL) challenge for 24 h. (A, B) Splenic lymphocytes were stained with the antibody of CD4^+^CD25^+^Foxp3^+^ (Treg) and analysed cell population by flow cytometry. (C, D) Flow cytometry analysed the proportion of CD3^+^CD4^+^IL‐17^+^ (Th17) cells in vitro. (E,F and G,H) The populations of CD3^+^CD4^+^IFNγ^+^ (Th1) cells and CD3^+^CD4^+^IL‐4^+^ (Th2) cells were tested by flow cytometry after SEA (25 μg/mL) treatment. Data were representative one experiment from two replicate experiments each with n = 4 mice per group. Data were presented as mean ± SEM. *P < .05; **P < .005; ***P < .001

To confirm the potential immunoregulatory role of PAK1, we further stimulated splenocytes from WT and PAK1‐deficient mice with schistosome soluble egg antigen (SEA) in vitro. Results showed that Treg cells were increased in the PAK1‐deficient splenocytes when compared with WT splenocytes in the presence of SEA (Figure 3A‐B); however, Th17 cells significantly declined in the splenocytes from PAK1‐deficient mice when compared with SEA‐treated WT splenocytes (Figure 3C‐D). The proportions of Th1 and Th2 cells were undifferentiated between WT and PAK1‐deficient group in the presence of SEA (Figure 3E‐F, G‐H).

### 3.4 PAK 1 induces Th17 cell differentiation through activation of macrophages

**Figure 4:**
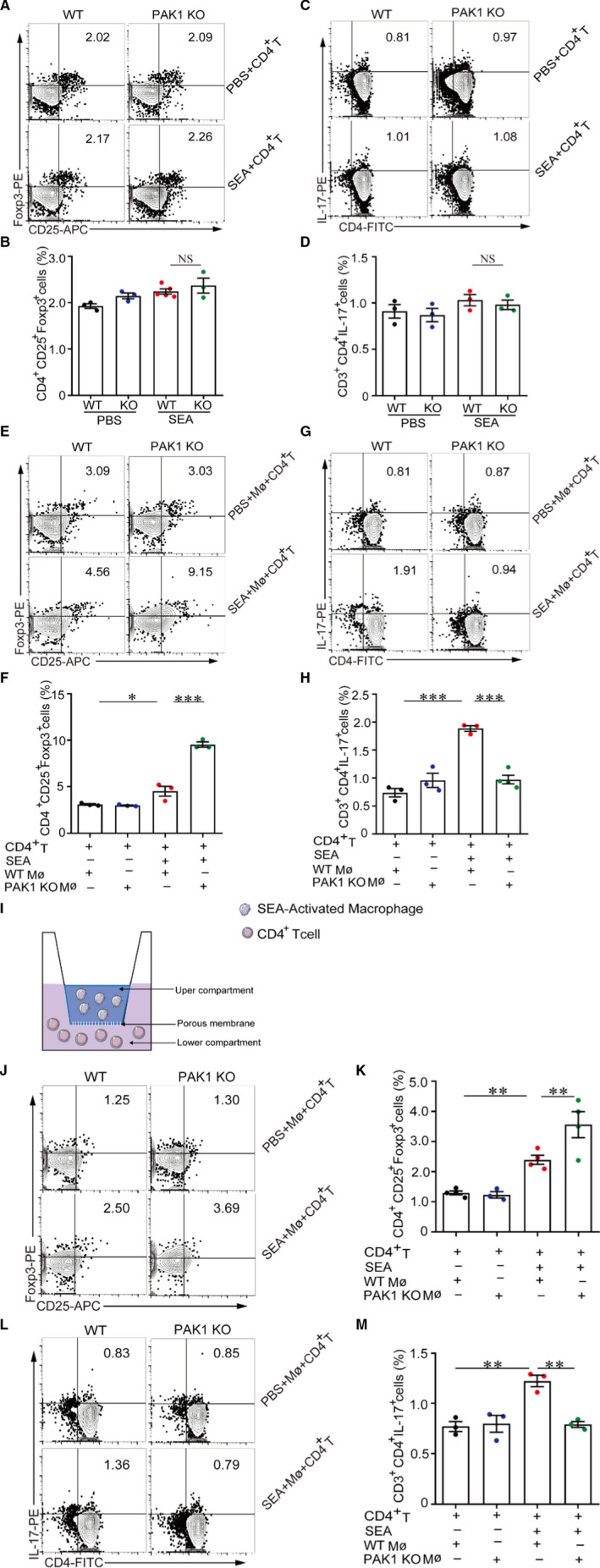
PAK1 regulates CD4^+^ T cells differentiation through activations of macrophages. (A,B, C,D) Purified CD4^+^ T cells (1 × 10^6^) were cultured in 6‐well plate and stimulated with SEA (25 μg/mL) for 24 h directly, CD4^+^CD25^+^ Foxp3^+^ (Treg) and CD3^+^CD4^+^IL‐17^+^ (Th17) cells were analysed via flow cytometry. (E,F and G,H) The peritoneal macrophages (5 × 10^5^) were stimulated with SEA (25 μg/mL) for 24 h and further co‐cultured with CD4^+^ T cells (1 × 10^6^) in 6‐well plate for 24 h, CD4^+^CD25^+^ Foxp3^+^ (Treg) and CD3^+^CD4^+^IL‐17^+^ (Th17) cells were measured by flow cytometry. (I) A schematic diagram of transwell assay. CD4^+^ T cells were seeded in the lower chamber in a 12‐well transwell with 0.4 μm inserts. SEA‐activated peritoneal macrophages were added into the upper chamber. (J,K and L,M) CD4^+^ T cells (1 × 10^6^) co‐cultured with SEA‐activated peritoneal macrophages (5 × 10^5^) in transwell system for 24 h, the proportions of CD4^+^CD25^+^ Foxp3^+^ (Treg) and CD3^+^CD4^+^IL‐17^+^ (Th17) cells were analysed by flow cytometry. Data were shown as mean ± SEM, n = 3. *P < .05; **P < .005; ***P < .001

To determine whether the alteration in the Th17/Treg balance in PAK1‐deficient mice was CD4^+^ T cells autonomous, CD4^+^ T cells from the spleen of normal WT and PAK1‐deficient mice were differentiated in vitro either in Treg or in Th17 cells after stimulation by SEA (Figure S4). Results showed that there were no significant changes in the percentages of Treg and Th17 cells (Figure 4A‐B, C‐D), suggesting that PAK1 in CD4^+^ T cells can not affect their differentiation under the egg antigen stimulation.

Macrophages, which have been reported to involve in the pathogenesis of granuloma formation, play a critical role in the initiation and regulation of adaptive immunity during *S* *japonicum* infection.^35^, ^36^, ^37^ To investigated whether PAK1 in macrophages could regulate CD4^+^ T‐cell differentiation, normal WT or PAK1‐deficient macrophages were stimulated with SEA and then co‐cultured with normal WT CD4^+^ T cells in vitro. Results showed that CD4^+^ T cells were preferentially differentiated towards Treg cells when exposed to SEA‐treated PAK1‐deficient macrophages compared with SEA‐treated WT macrophages (Figure 4E‐F); however, in vitro treatment of PAK1‐deficient macrophages derived from uninfected mice with SEA inhibited Th17 cell differentiation (Figure 4G‐H).

To further differentiate the effects of physical contact *vs*. soluble factors of WT or PAK1‐deficient macrophages on CD4^+^ T cells differentiation, we conducted a transwell co‐culture system in which SEA‐stimulated macrophages and normal CD4^+^ T cells were grown separated by a membrane via interactions with soluble factors (Figure 4I), results showed that SEA‐stimulated PAK1‐deficient macrophages still promoted relatively higher levels of Treg cells but lower Th17 cells when compared with WT macrophages (Figure 4J‐K, L‐M). Taken together, this observation suggests that PAK1 deficiency can influence the Treg/Th17 cell differentiation in an indirect way.

### 3.5 PAK1 promotes IL‐6 expression through IRF1/p65 axis in macrophages

**Figure 5:**
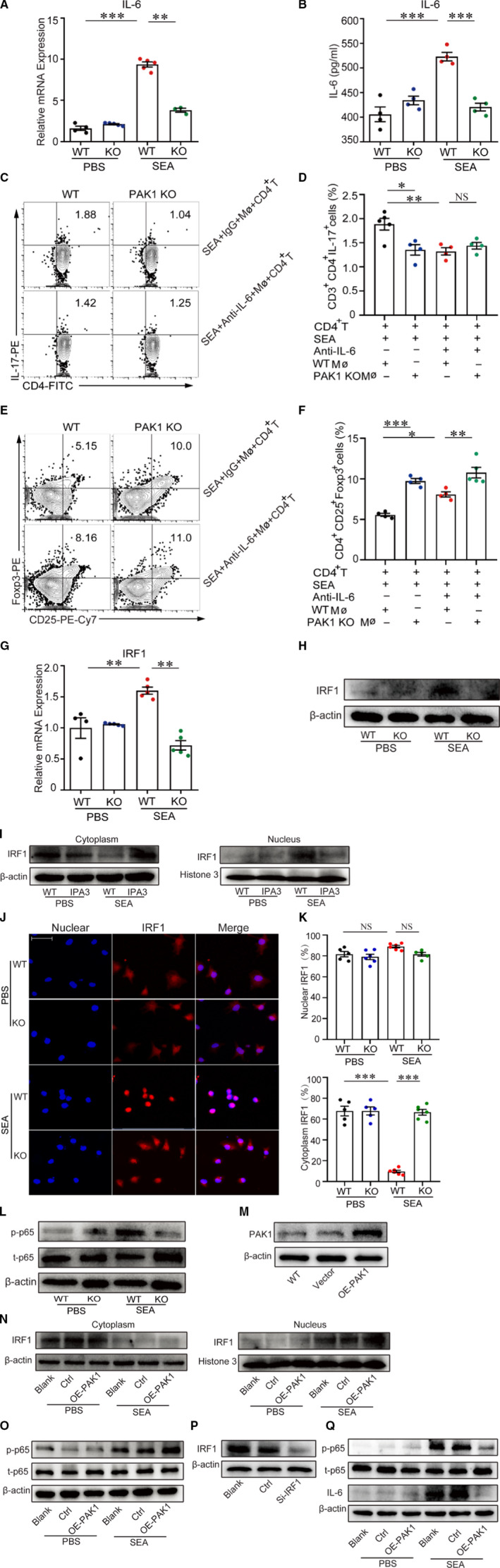
PAK1 promotes Th17 differentiation by inducing IL‐6 expression in macrophages. Peritoneal macrophages (5 × 10^5^) were separated from WT or PAK1‐deficient mice and stimulated with SEA (25 μg/mL) for 24 h in a 6‐well plate, the mRNA of IL‐6 was analysed via real‐time quantitative PCR (A), and cellular supernatant was collected and measured the level of IL‐6 via ELISA (B), n = 5. (C‐D, E‐F) SEA‐activated WT or PAK1‐deficient macrophages (5 × 10^5^) co‐cultured with WT CD4^+^ T cells (1 × 10^6^) in the presence or absence of mouse IL‐6‐neutralizing antibody (5 μg/mL) or IgG controls for 24 h, flow cytometry analysed the proportion of CD3^+^CD4^+^IL‐17^+^ (Th17) and CD4^+^CD25^+^ Foxp3^+^ (Treg) cells. (G, H) The mRNA and protein levels of IRF1 was identified by real‐time quantitative PCR and Western blot, n = 5. (I) We stimulated RAW264.7 with IPA3, which was an inhibitor of PAK1. Western blot analysis of IRF1 protein from cytoplasm and nucleus in RAW264.7 after SEA (25μg/mL) stimulation for 24 h. (J‐K) Immunofluorescence of IRF1 (Red) in primary peritoneal macrophages was observed in the presence of PBS or SEA (25 μg/mL) (Scale bars, 50 μm), n = 8. Data were representative of one experiment from two replicate experiments. Data were presented as mean ± SEM. *P < .05; **P < .005; ***P < .001. (L) The peritoneal macrophages (5 × 10^5^) were treated with PBS or SEA (25 μg/mL) for 24 h, phosphorylated‐p65 and total p65 detected via Western blotting. (M) RAW264.7 cells were transfected with the plasmid overexpressing PAK1, and the protein of PAK1 was analysed by Western blotting. (N) We separated the protein from cytoplasm and nucleus in WT and PAK1‐overexpressed RAW264.7 after SEA (25 μg/mL) stimulation for 24 h, and IRF‐1 expression was analysed by Western blotting. (O) Western blot analysed the expression of total p65 and phosphorylated‐p65 protein in WT and PAK1‐overexpressed RAW264.7 incubated with SEA (25 μg/mL) for 24 h. (P) Macrophages were transfected with siRNA targeting IRF1 (si‐IRF1), and the protein of IRF1 was analysed by Western blot. (Q) Western blot analysed the protein levels of IL‐6 and phosphorylated p65 in WT and IRF1 knockdown macrophages in the presence or absence of SEA. Data were one representative experiment from two experiments with similar results (I, L‐Q)

IL‐6 has been reported to mediate CD4^+^T cells differentiate into Th17 subset through activation of RORγt.^38^ In order to investigate the mechanism of PAK1‐mediated regulation of CD4^+^T‐cell differentiation, we detected the expression of IL‐6 in the primary macrophages from WT and PAK1‐deficient mice. Results showed that both mRNA and protein levels of IL‐6 were significantly decreased in SEA‐treated PAK1‐deficient macrophages or cell culture supernatants when compared with SEA‐stimulated WT macrophages (Figure 5A, B). Also, the M1 macrophages‐related genes (TNF‐α, IL‐12 and iNOS) were significant decreased in PAK1‐deficient macrophages after SEA stimulation when compared with WT macrophage, while the M2 macrophages‐related gene (Arg‐1) was increased (Figure S5); however, the mRNA levels of IL‐10 and IL‐13 in PAK1‐deficient macrophages were similar in the WT macrophages after SEA stimulation (Figure S6). To investigated whether the PAK1‐mediated regulation of pathogenic Th17 cell responses is dependent on IL‐6 production, the mouse IL‐6‐neutralizing antibody was used and results showed that neutralization of IL‐6 in SEA‐stimulated WT macrophages dampened the induction of Th17 (Figure 5C‐D) but promoted Treg cell response (Figure 5E‐F). Therefore, these results show that the production of IL‐6 in macrophages is necessary for the PAK1‐mediated Th17 response.

To further examined the molecular mechanism underlying the observed IL‐6 induction in PAK1‐deficient macrophages, we predicted the mediator of IL‐6 on the website (https://genome.ucsc.edu/) and found that interferon regulatory factor 1 (IRF‐1) was a vital regulatory factor for IL‐6 production (Figure S7). Thus, we compared the expression of IRF1 in SEA‐stimulated WT and PAK1‐deficient macrophages. Results showed that the mRNA and protein levels of IRF1 were decreased in the SEA‐stimulated PAK1‐deficient macrophages when compared with WT macrophages (Figure 5G, H, Figure S8). We further analysed the location and expression of IRF1 in the cytoplasm and nucleus of macrophages by Western blotting and confocal microscopy. SEA‐treated WT macrophages resulted in increased more nuclear protein amount of IRF1 when compared with untreated macrophages; however, less IRF1 nuclear translocation in SEA‐treated PAK1‐deficient macrophages was observed by Western blot (Figure 5I, Figure S8). Similarly, the cytoplasmic IRF1 was remarkably higher in SEA‐stimulated PAK1‐deficient macrophages than SEA‐stimulated WT macrophages (Figure 5J‐K), indicating that loss of PAK1 inhibits translocation of IRF1 from the cytoplasm into the nucleus. Together, these results indicate that PAK1 promoted IRF1 nuclear translocation and IL‐6 production in macrophages.

Previous study had confirmed that IRF1 played a vital role in promoting the activation of NF‐κB signalling,^39^ which has an important role in regulating immune response and inflammation diseases.^40^ We found that the phosphorylated‐p65 but not total p65 was significantly reduced in PAK1‐deficient macrophages when compared with SEA‐stimulated WT macrophages (Figure 5L, Figure S8). In addition, overexpression of PAK1 in macrophage cell line RAW264.7 (Figure 5M, Figure S8) resulted in dramatically increased IRF1 nuclear transcription and the expression of phosphorylated‐p65 after SEA stimulation (Figure 5N, O, Figure S8). To further determine the contribution of IRF1 for the IL‐6 induction, siRNA was used to inhibit IRF1 expression in SEA‐stimulated macrophages (Figure 5P, Figure S8) and results showed that knockdown of IRF1 significantly reduced the expression of IL‐6 and phosphorylated p65 in SEA‐treated macrophages (Figure 5Q, Figure S8). Altogether, these results suggest that PAK1 is essential for the activation of IRF1/NF‐κB pathway in macrophages.

## 4 DISCUSSION

Although it has been reported that PAK1 has an important role in immune responses and inflammatory disease,^14^, ^41^ the function of PAK1 in regulating the differentiation of CD4^+^ T cells has remained unclear. Our findings firstly indicated that PAK1 can facilitate CD4^+^ T cells to differentiate towards Th17 cells but suppress Treg cells by regulating the secretion of IL‐6 from macrophages in a murine *S* *japonicum‐*infected model. Furthermore, we found that PAK1 can promote the nuclear translocation of IRF1 and induce IL‐6 expression through the NF‐κB‐dependent pathway.

Schistosomiasis was one important parasitic disease that affects more than 250 million people worldwide.^42^ The main immunopathology in schistosomiasis japonica was the egg induced granulomatous inflammatory response and fibrosis in the liver and intestines, which was the primary cause of mortality associated with this chronic disease. In this study, our findings indicated that the overexpressed PAK1 was associated with serious hepatic pathology during *S* *japonicum* infection. And consistently, PAK1 deficiency resulted in reduced granuloma size and fibrosis in the livers from *S* *japonicum‐*infected mice. Therefore, we inferred that PAK1 may play a vital role in promoting hepatic immunopathology.

The subsets of CD4^+^ T cells were major participants in the development of immunopathology during *S* *japonicum* infection.^17^ It is reported that Th1 and Treg cells contributed to suppress liver pathology,^43^, ^44^ while Th2 and Th17 aggravated the granulomatous and fibrosis in schistosomiasis.^20^, ^21^, ^45^ Considering the potential role of PAK1 in the regulation of immune response, we supposed that PAK1 relieved immunopathology by affecting the differentiation of CD4^+^ T cells. Our results showed that PAK1 deficiency can up‐regulate Treg cells, which were critical for attenuating the local inflammatory response and facilitated a significant improvement of immunopathology during schistosome infection.^21^, ^43^ As a crucial family of serine/threonine kinases, PAK1 affects plenty of cellular processes and served an important role in different biological functions in diverse diseases, including cell immigration, invasion, growth and apoptosis in various cancers.^46^ It is reported that genetic deletion of PAK1 in mice significantly attenuates circulating IL‐6 and MCP‐1 levels in atherosclerosis‐prone apolipoprotein E‐deficient (ApoE^‐/‐^) mice,^47^ PAK1 was also found to induce the secretion of IL‐1β by activating caspase‐1 in macrophages,^48^ suggested the potential role of PAK1 in the regulation of inflammation.^49^, ^50^ In addition, PAK1 also increased phagocytosis of gitter cell by elevating NADPH in the stimulation of N‐formyl methionyl‐leonyl‐phenylalanine (fMLP).^51^ Notably, this is the first work to examine the effects of PAK1 on the regulation of adaptive immune response during pathogen infection.

As one critical antigen‐presenting cell, macrophage has a pivotal role in regulating immunopathology in schistosomiasis japonica. During the acute stage of schistosome infection, the soluble worm antigens (SWA) induced the activation of pro‐inflammation macrophages (M1 macrophage), characterized by the elevated interleukin‐12 (IL‐12), IL‐6 and tumour necrosis factor‐alpha (TNF‐α), which are critical for direct host defence against pathogens.^16^ After the egg production, the soluble egg antigens (SEA) induced M2‐dominated macrophages, which are important for the maintenance of hepatic granulomas and host survival.^52^, ^53^ Interesting, several studies confirmed that M1‐related molecules were also activated after schistosoma spawns, as evidenced by enhanced M1‐related markers, such as IL‐12, TNF‐α, CXCL9 and CXCL11, in SEA‐stimulated macrophages.^19^, ^54^ Study has showed that PAK1 can facilitate M1 macrophage polarization and induce the secretion of TNF‐α.^55^ In our study, we demonstrated PAK1 facilitates CD4^+^ T cells to differentiate into Th17 cells dependent on the inflammatory cytokines produced by M1‐like macrophage.

It is reported that IL‐6 contributes to the Th17 cells differentiation,^38^ we found that the expression of IL‐6 in PAK1‐deficient macrophage was significantly decreased, suggesting that macrophage‐derived IL‐6 is necessary for Th17 cell differentiation. Recently, transcription factor IRF1 has been reported to play a vital role in the host defence against pathogens and the development of the immune system.^56^ The expression of IRF1 was increased in response to IFN‐γ and LPS stimulation and IL‐6 was secretion in macrophages, while knockdown of IRF1 led to significantly decreased expression of IL‐6,^57^ indicating that IRF1 plays an essential role in the IL‐6 production. We speculated that activation of IRF1 might be involved in PAK1‐mediated IL‐6 production through online prediction. Indeed, we confirmed that IRF1 nuclear translocation was induced by PAK1 using PAK1‐deficient macrophages, although we have not examined the exact binding sites of IL‐6 for IRF1. Previous study showed that PAK1 can activate IL‐6 expression via JaK2 activation in cancer stem cell,^58^ whether JaK2 involved in the PAK1‐mediated IL‐6 production needs further study to confirm.

The IRF1 has been reported to play an essential role in promoting NF‐κB activation and inducing IL‐6 production.^39^ To clarify whether PAK1‐mediated IRF1 activation subsequently affects NF‐κB activation,^59^ phosphorylated p65 was detected and results showed that the level of phosphorylated p65 was decreased in PAK1‐deficient group in the presence of SEA, suggesting that PAK1 is essential for the activation of IRF1/NF‐κB signalling in macrophages. As PAK1 is effector molecules of small GTPases,^60^ whether PAK1‐mediated IRF1 signalling is dependent on GTPases or not needs further investigation.

Collectively, our data demonstrate that macrophage PAK1 facilitates Th17 cell differentiation via IL‐6 production and further promotes hepatic immunopathology. Our data analysed the mechanism of CD4^+^ T‐cell differentiation and interaction with macrophages in disease states and provide a framework for understanding the complexity of CD4^+^ T‐cell differentiation.

## CONFLICTS OF INTEREST

The authors have no conflict of interest to declare.

## AUTHOR CONTRIBUTION

**Hao Chang:** Data curation (lead); Methodology (lead); Writing‐original draft (lead). **Kaiyue He:** Software (equal). **Chen Li:** Data curation (equal); Methodology (equal). **Yangyue Ni:** Data curation (equal). **Lin Chen:** Methodology (equal). **Min Hou:** Data curation (equal). **Maining Li:** Data curation (equal). **Zikai Zhou:** Methodology (equal); Resources (equal). **Zhipeng Xu:** Funding acquisition (lead); Project administration (equal); Supervision (lead); Writing‐original draft (lead); Writing‐review & editing (lead). **Minjun Ji:** Project administration (equal); Supervision (lead); Writing‐review & editing (equal).

## Supporting information

Supplementary Material

## Data Availability

All data of this study are available from the first author upon reasonable request.
