# Supplementary material for: P21 activated kinase‐1 (PAK1) in macrophages is required for promotion of Th17 cell response during helminth infection
Source: J Cell Mol Med. 2020 Oct 30;24(24):14325–38. doi: 10.1111/jcmm.16050 (PMC7753984; doi:10.1111/jcmm.16050)
Supplement: Supplementary file 1 — Supplementary Material [file JCMM-24-14325-s001.docx]

**Supporting information**

**Table 1**

| Gene | | Forward (5’-3’) | Reverse (5’-3’) |
| --- | --- | --- | --- |
| GAPDH | AACTTTGGCATTGTGGAAGG | | GGATGCAGGGATGATGTTCT |
| IL-4 | GGTCTCAACCCCCAGCTAGT | | GCCGATGATCTCTCTCAAGTGAT |
| IL-13 | GCCAGCCCACAGTTCTAC | | GAGATGTTGGTCAGGGAAT |
| Arg-1  iNOS  TNF-α  IL-12 | CAGAAGAATGGAAGAGTCAG  GCCACCAACAATGGCAACA  CATCTTCTCAAAATTCGAGTGACAA  GAGGATACCACTCCCAACAGACC | | CAGATATGCAGGGAGTCACC  CGTACCGGATGAGCTGTGAATT  TGGGAGTAGACAAGGTACAACCC  AAGTGCATCATCGTTGTTCATACA |
| IL-10 | ACTTTAAGGGTTACTTGGGTTGC | | ATTTTACAAGGGGAGAAATCG |
| PAK1  PAK2  PAK3  PAK4 | GAAACACCAGCACTATGATTGGA  AACGGAGAGCTAGAAGACAAGC  CTGAGGATGAACAGTAACAACCG  GCTCCAACTCCCTACGCAG | | GAAACACCAGCACTATGATTGGA  TGGAACAGAAGGCAAAGGTTT  CTGGGAAGATAGAGCGAAGCC  TGGACCATCCCTCGAAGATTT |
| IL-6 | GAGGATACCACTCCCAACAGACC | | AAGTGCATCATCGTTGTTCATACA |
| IRF1 | ATGCCAATCACTCGAATGCG | | TTGTATCGGCCTGTGTGAATG |


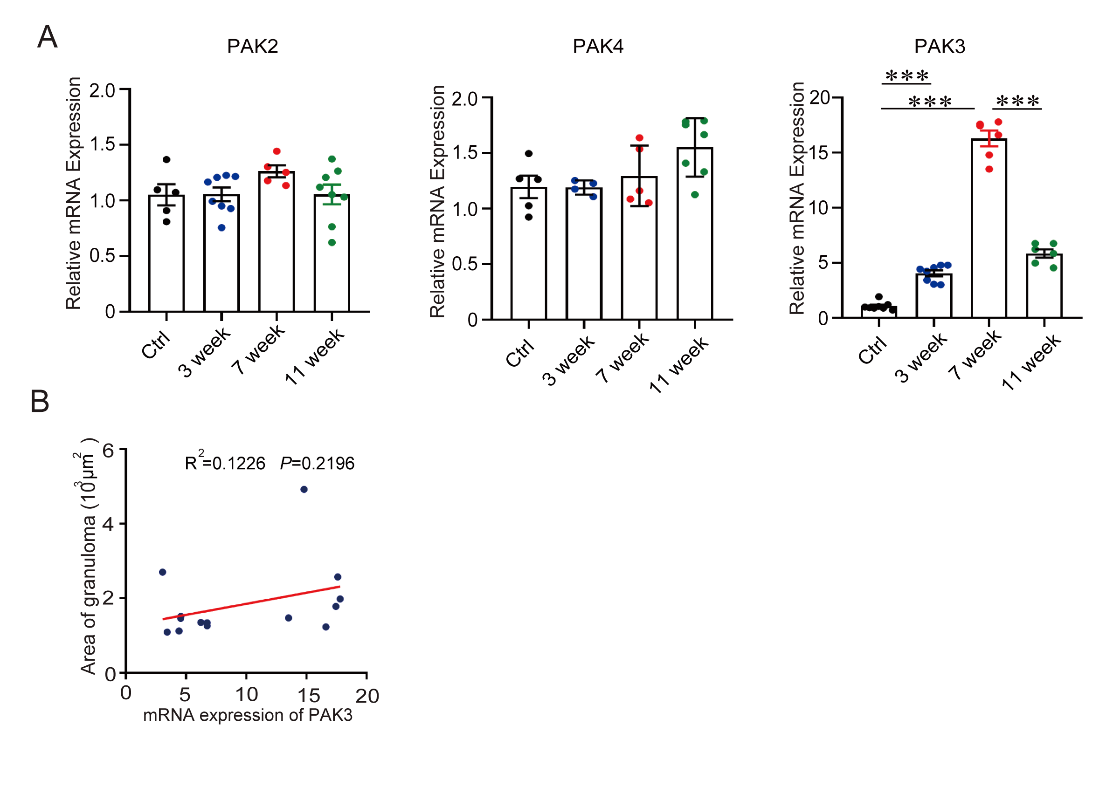


**Supplementary Figure 1:** The effects of *S. japonicum* infection on the expression pattern of PAKs (PAK2, PAK3, and PAK4).


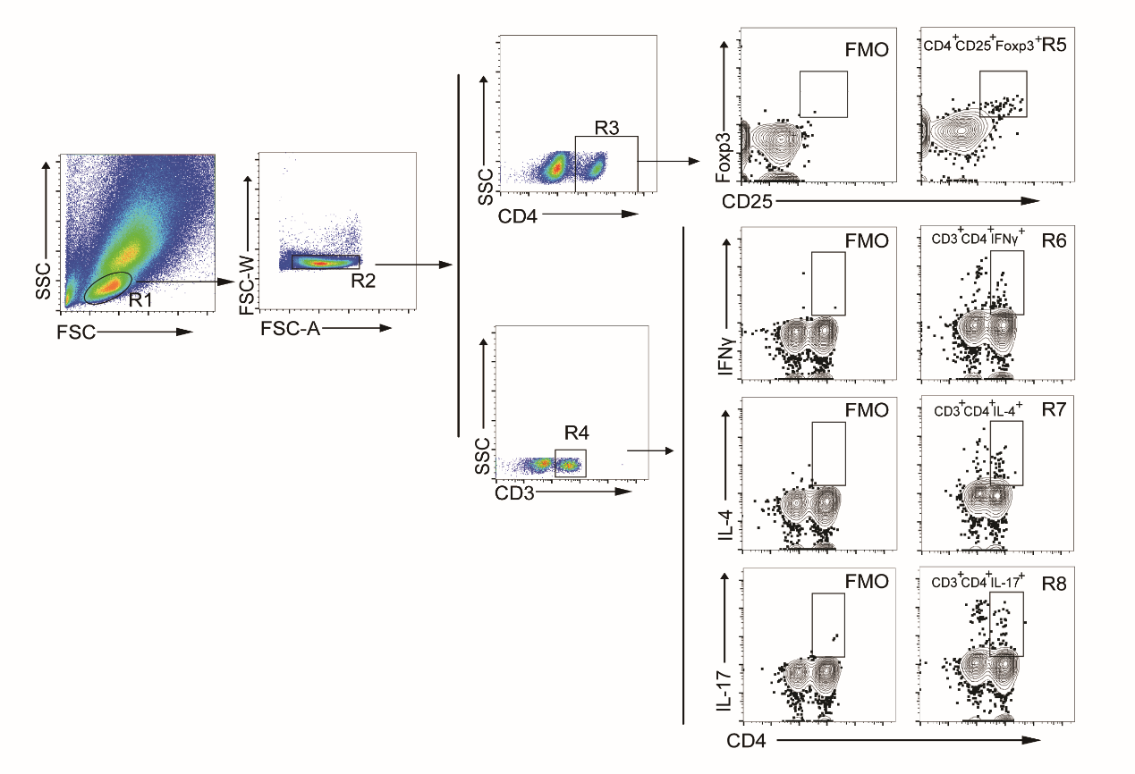


**Supplementary Figure 2:** FACS gating strategies.


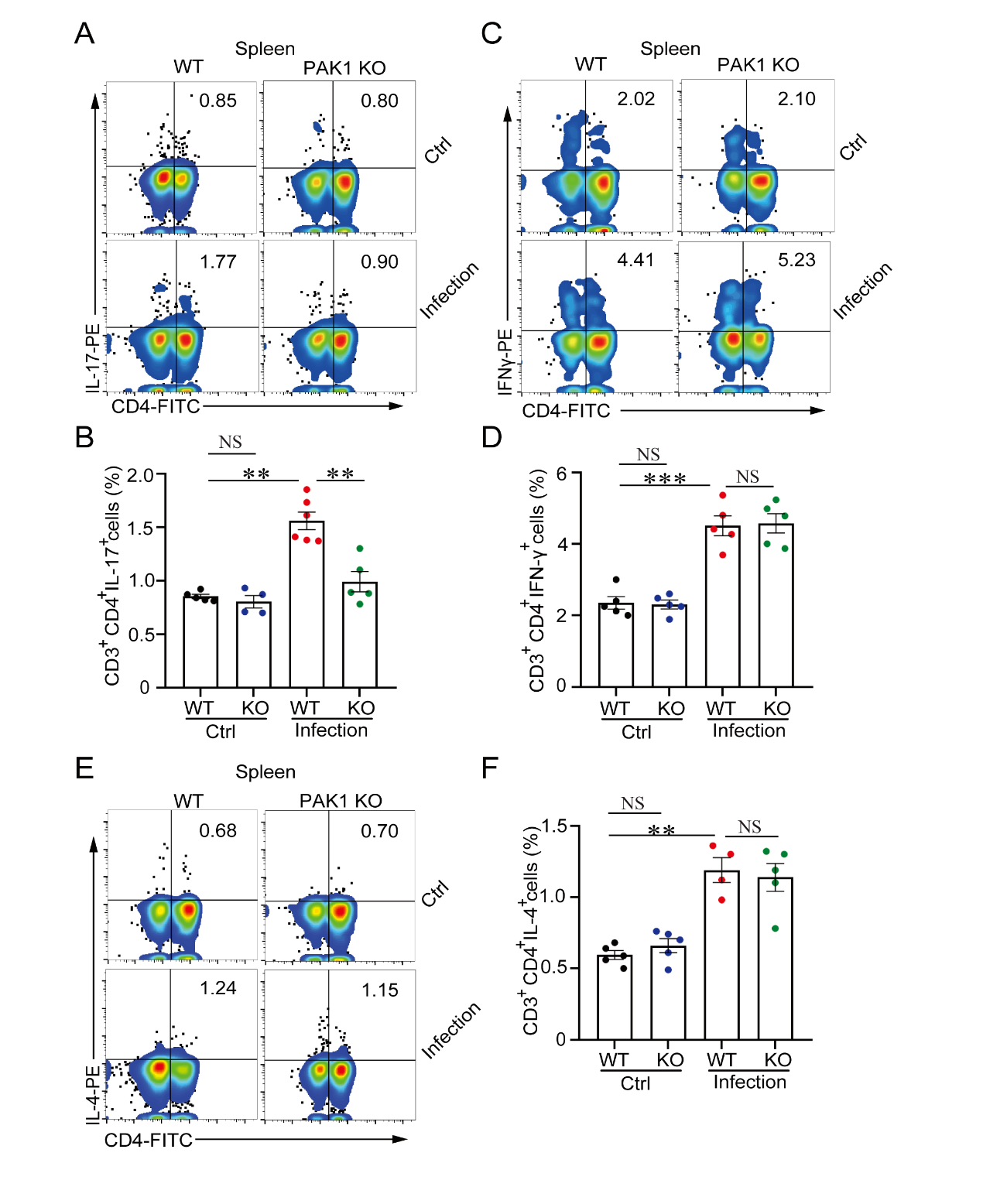


**Supplementary Figure 3:** The changes of CD4^+^ T cell subsets in the spleen after *S*. *japonicum* infection.


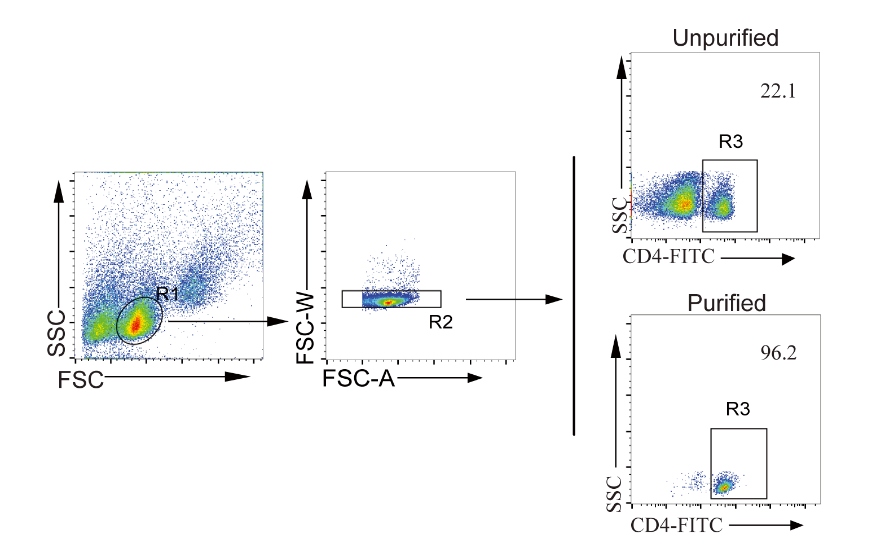


**Supplementary Figure 4:** The purity of CD4^+^T cells.


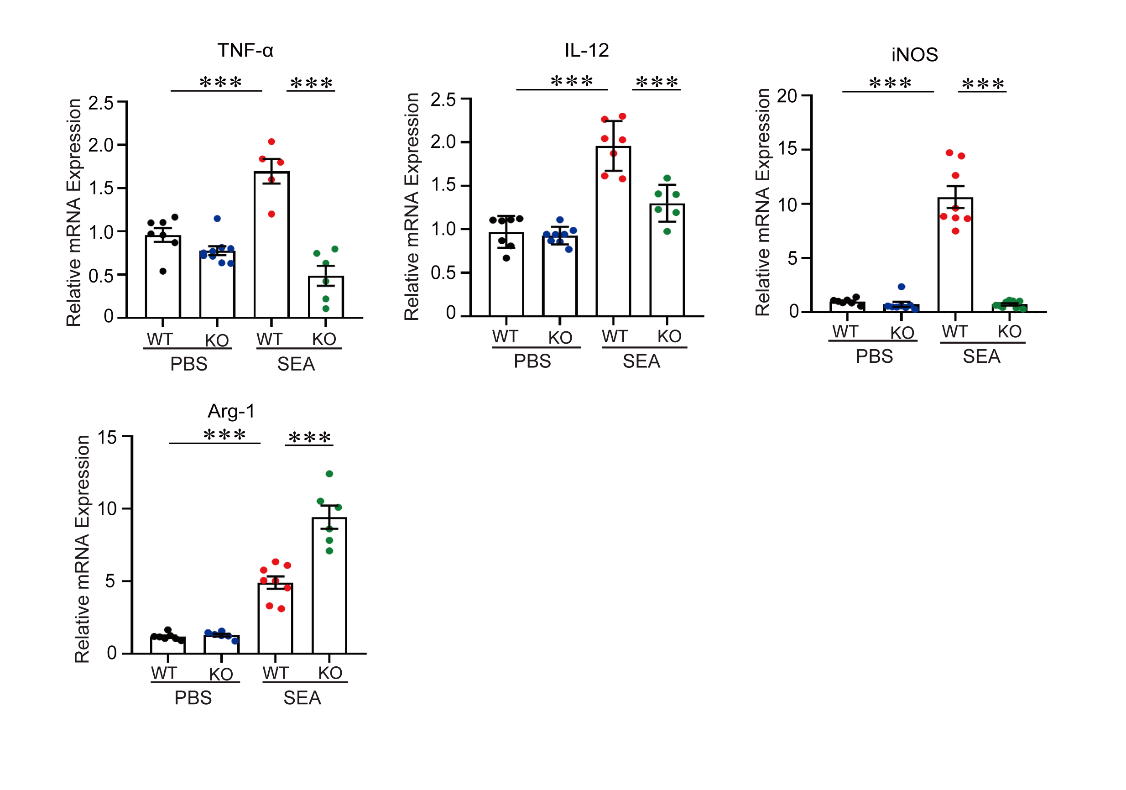


**Supplementary Figure 5:** PAK1 deficiency can induce M2-like phenotype in macrophages.


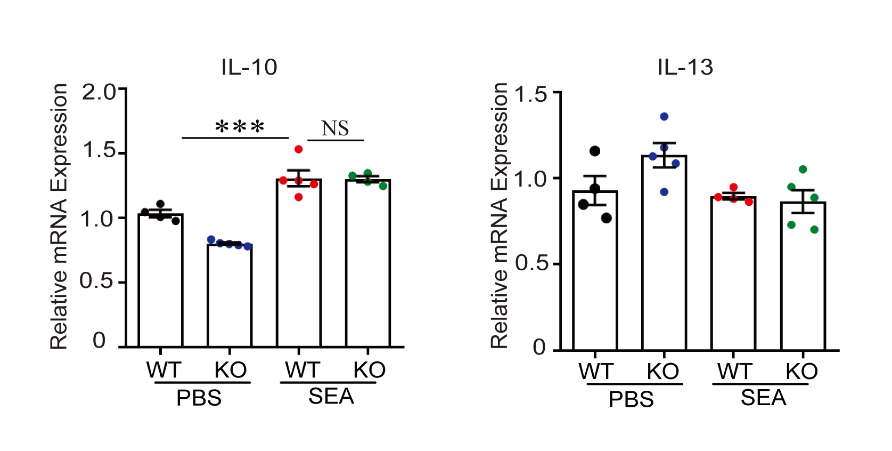


**Supplementary Figure 6:** PAK1 can affect the secretion of anti-inflammatory and pro-inflammatory cytokines in macrophages.

**
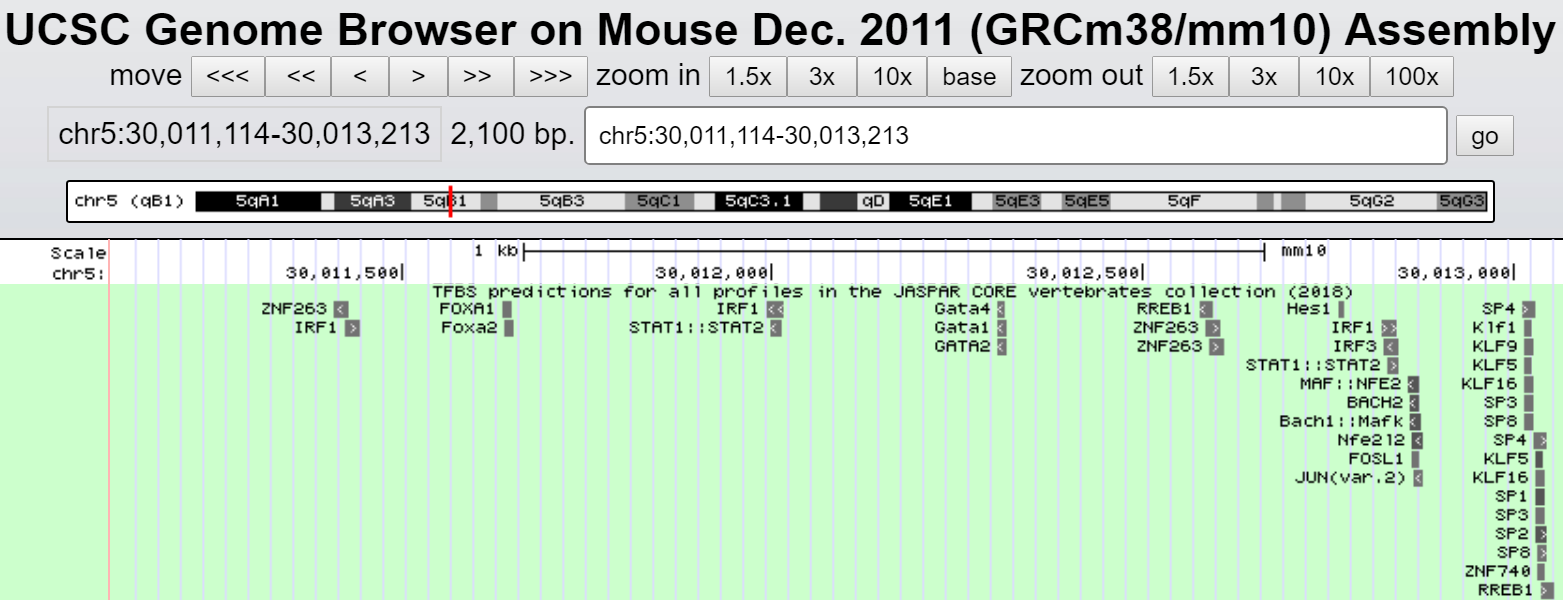
**

**Supplementary Figure 7:** Interferon regulatory factor 1 (IRF1) is a vital regulatory factor for IL-6 production.

**Supplementary Figure 8:** The uncropped western blots in the main figures.


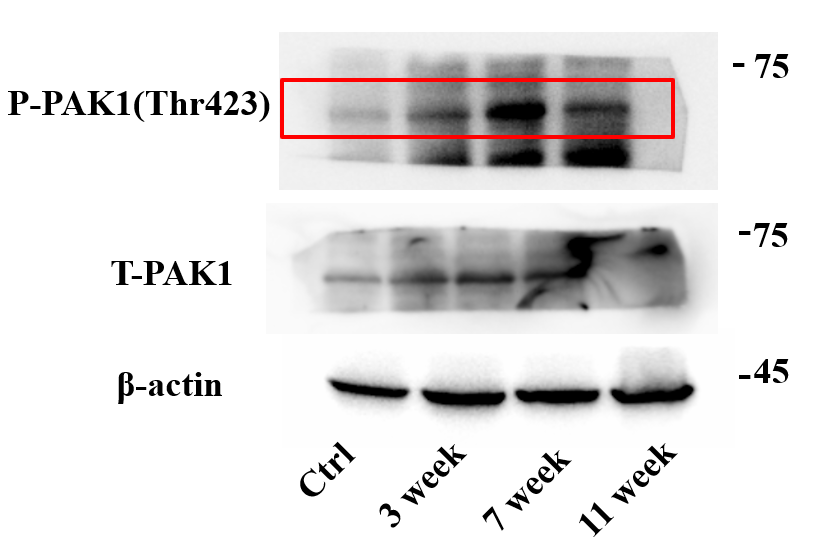


Uncropped western blots related to Figure 1B in the manuscript. The dynamic protein expression of phosphorylated PAK1 (Thr423) via western blot in the livers from *S. japonicum*-infected mice was conducted.


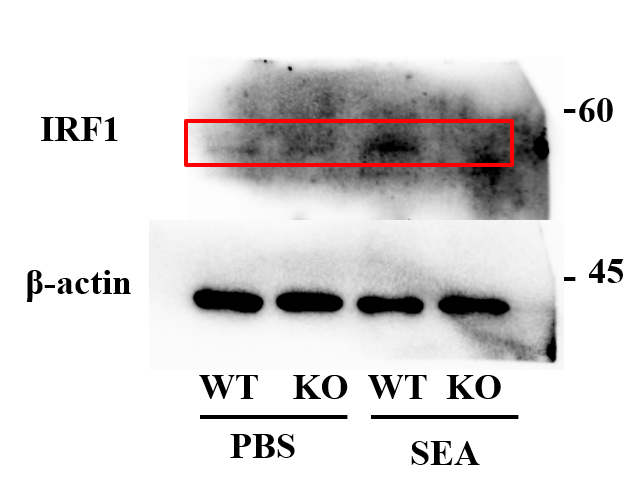


Uncropped western blots related to Figure 5H in the manuscript. The protein levels of IRF1 was identified by western blot in PAK1-deficient macrophages and WT macrophages.


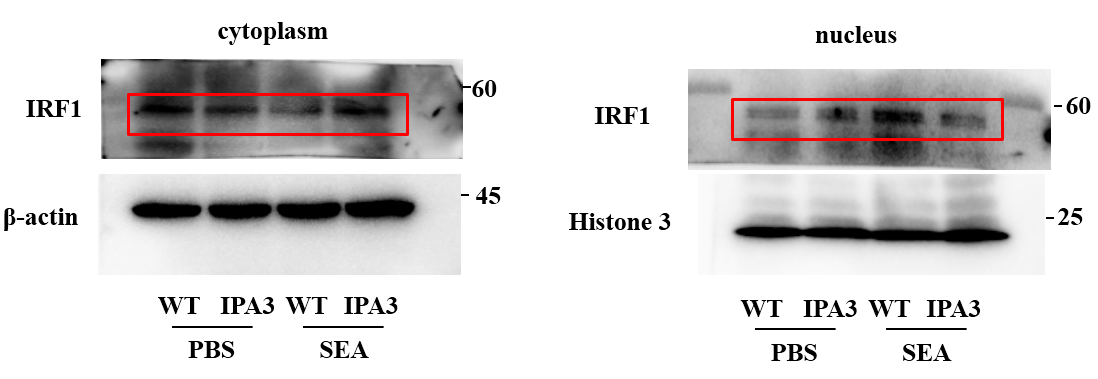


Uncropped western blots related to Figure 5I in the manuscript. Western blot analysis of IRF1 protein from cytoplasm and nucleus in RAW264.7 after SEA (25μg/ml) stimulation for 24 h.


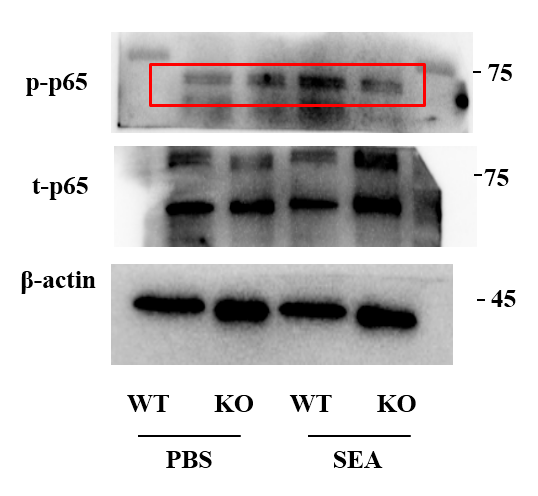


Uncropped western blots related to Figure 5L in the manuscript. The peritoneal macrophages (5×10^5^) were treated with PBS or SEA (25 μg/ml) for 24 h, phosphorylated-p65 and total p65 detected via Western blotting.


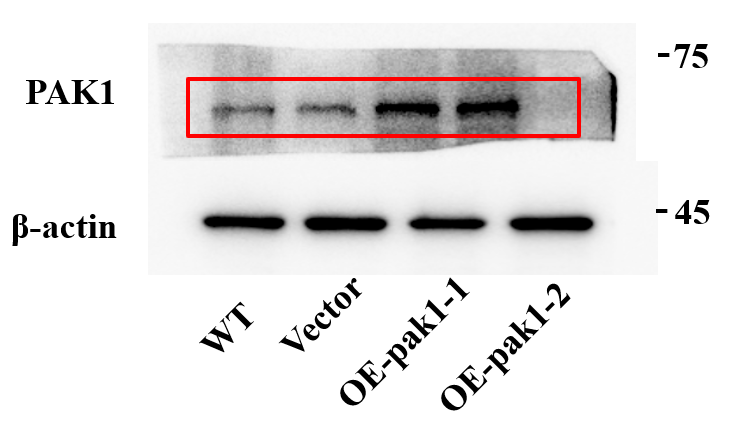


Uncropped western blots related to Figure 5M in the manuscript. RAW264.7 cells were transfected with the plasmid overexpressing PAK1 and the protein of PAK1 was analyzed by western blotting.


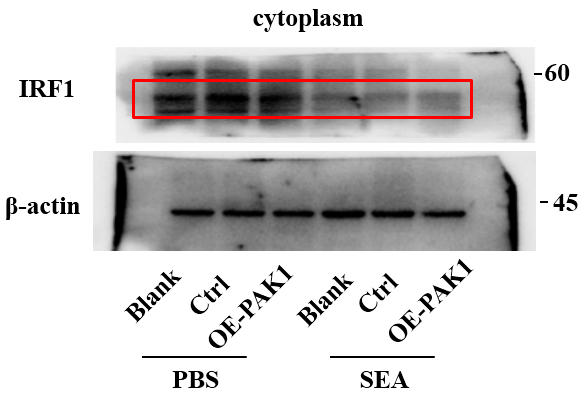


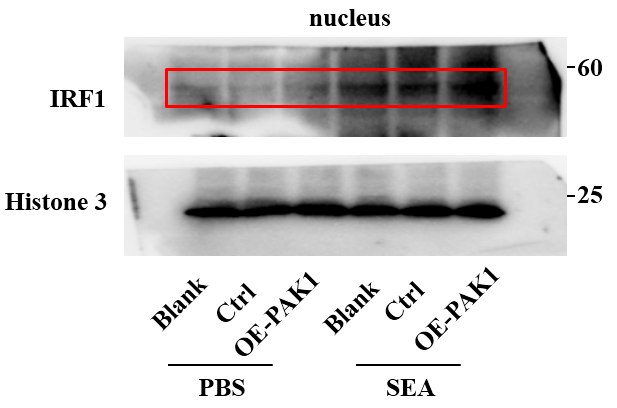


Uncropped western blots related to Figure 5N in the manuscript. We separated the protein from cytoplasm and nucleus in WT and PAK1-overexpressed RAW264.7 after SEA (25 μg/ml) stimulation for 24 h, and IRF-1 expression was analyzed by western blotting.


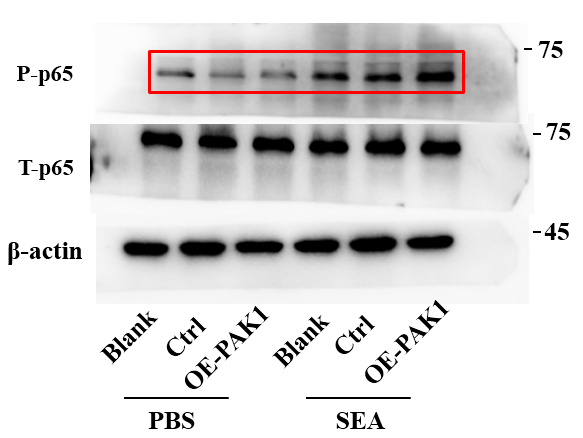


Uncropped western blots related to Figure 5O in the manuscript. Western blot analyzed the expression of total p65 and phosphorylated-p65 protein in WT and PAK1-overexpressed RAW264.7 incubated with SEA (25 μg/ml) for 24 h.


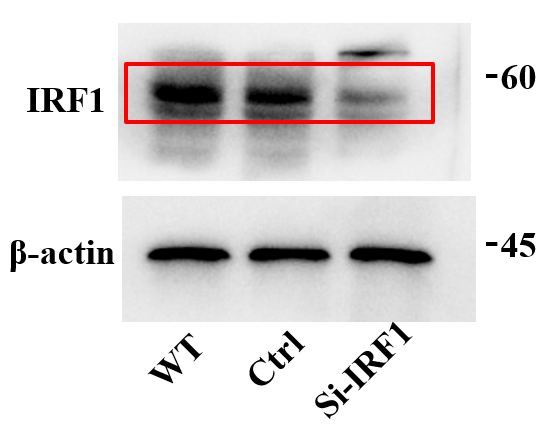


Uncropped western blots related to Figure 5P in the manuscript. Macrophages were transfected with siRNA targeting IRF1 (si-IRF1) and the protein of IRF1 was analyzed by western blot.


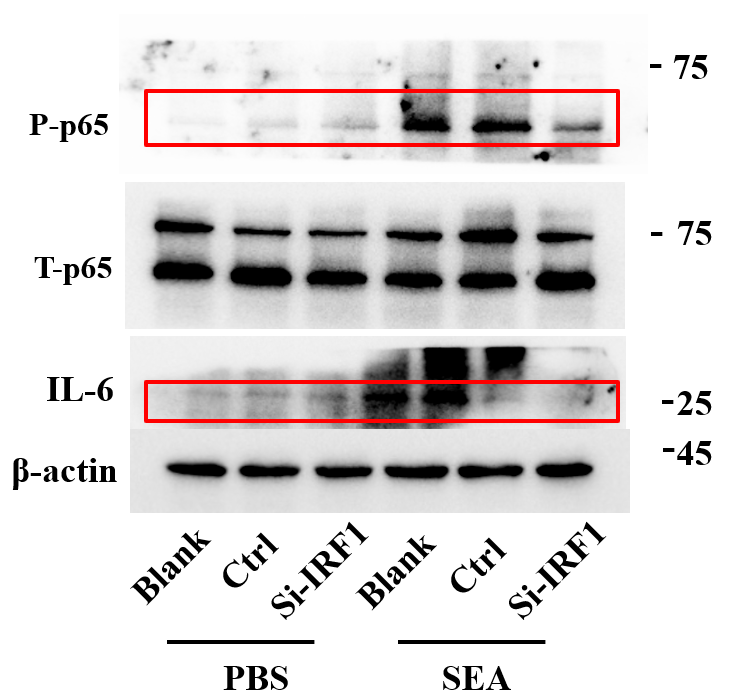


Uncropped western blots related to Figure 5Q in the manuscript. Western blot analyzed the protein levels of IL-6 and phosphorylated p65 in WT and IRF1 knockdown macrophages in the presence or absence of SEA.
